# Consensus on relevant point-of-care ultrasound skills in General Practice: a two-round French Delphi study

**DOI:** 10.1186/s12909-024-05072-3

**Published:** 2024-03-26

**Authors:** Louis Camard, Roxane Liard, Sophie Duverne, Gladys Ibanez, Mariela Skendi

**Affiliations:** 1 Department of General Practice, Faculty of Medicine, Sorbonne University, Paris, France; 2 Department of Adult Radiology, Necker Hospital, Paris, France; 3 Pierre-Louis Institute of Epidemiology and Public Health, Paris, France

**Keywords:** Point-of-care systems, Ultrasonography, General practitioner, Family physician, Primary care, General practice, Delphi study, Consensus

## Abstract

**Context:**

Point-of-Care Ultrasound (POCUS) has become an important tool in the clinical practice of many specialties, but its use and impact in General Practice in France remains to be explored.

**Objective:**

The objective of this study is to obtain a consensus among experienced French general practitioners on a list of relevant POCUS skills in General Practice in 4 anatomical regions.

**Method:**

We used a two-round Delphi method to obtain a consensus. An initial list of skills was drawn by conducting a literature review. To rate each skill, we used a nine-point Likert scale. An interactive meeting between experts took place between Delphi rounds. POCUS experts in General Practice were defined as general practitioners with theoretical training in ultrasound who regularly perform ultrasound, who have performed ultrasound for more than five years and/or are involved in providing ultrasound training.

**Results:**

11 French general practitioners screened 83 skills in 4 anatomical regions: abdominal, urogenital, vascular, gynecology and obstetrics. An agreement was obtained for 36 POCUS skills as to their appropriateness in General Practice. There were 17 skills with a strong appropriate agreement (100% of “7–9” ratings) and 19 skills with a relative agreement (100% of “5–9” ratings).

**Conclusion:**

These skills could serve as a basis for guidelines on the use and curriculum of POCUS in General Practice in France as well as in other countries with similar healthcare systems.

**Supplementary Information:**

The online version contains supplementary material available at 10.1186/s12909-024-05072-3.

## Introduction

The use of ultrasound discovered in the nineteenth century is progressing in different medical specialties [1]. This technique is not solely used as a complementary examination, but it has evolved into the clinical point-of-care tool it is today through miniaturization and cost reduction. However, it is a dynamic, operator-dependent imaging technique that requires specific knowledge and skills [2]. Its use is growing in General Practice and some skills seem relevant both in terms of validity and impact on patient care even if the training arrangements have still to be specified [3].

In 2017, *​​T*he *American Academy of Family Physicians (AAFP)* created curriculum guidelines for POCUS in graduate medical education [4]. Since 2020, several European studies have tried to describe a list of indications and applications in General Practice obtained by consensus of Scandinavian [5] and Spanish [6] general practitioners. In France, the *French Health Authority* conducted a literature review in 2022 that identified a lack of data on the use of POCUS by general practitioners [7]. It is necessary to have a framework of relevant POCUS skills in general practice, to precise the modalities of use, the clinical situations where they have an impact on patient care and their training requirements [7].

The objective of the study was to determine a consensus on relevant POCUS skills in General Practice, among experienced French general practitioners, on a list of relevant POCUS skills in General Practice in the following fields: abdominal and digestive, urogenital, vascular, gynecological and obstetrics.

## Methods

This study was conducted in France from November 2021 to July 2022. We used the Delphi method to ascertain the degree of agreement within a group of selected individuals. This is a qualitative method used to address subjects for which few studies are available and/or where there is a low level of evidence. It enables structured interaction between experts through several self-completed questionnaires and successive meetings. It follows three key principles: data anonymity, repeated collection of data with comments and data analysis with feedback given to the various participants [8–10].

The search for experts was carried out with the help of the French College of General Practice Ultrasound group, made up of 16 members, representing all French College of General Practice structures (unions and training organizations). The invitation email including a questionnaire on the characteristics of general practitioners practicing ultrasound was relayed by the members of the group to the 14 structures represented and distributed via the mailing lists or the website of each structure with a follow-up after 1 month. We do not know the total number of general partitioners (GP) addressed but the invitation email was sent to all GPs that were union members, that had participated in continuous medical education or were part of the network of "ECHO-MG", an ongoing study on POCUS in general practice [11]. The responses from interested physicians were analyzed and those who met the inclusion criteria were contacted.

The inclusion criteria for the experts were:

- to be a general practitioner in France;
- to practice ultrasound regularly: more than once a week and for more than two years;
- to have theoretical training in ultrasound: university program that includes theoretical courses (physics of ultrasound, normal and pathological findings) followed by hands-on internships in each area;
- to have practiced ultrasound for more than five years and/or be involved in providing ultrasound training.

### Delphi process

#### Questionnaire drafting phase

The initial questionnaire was created by the authors of this study. The documentary research strategy involved a review of international recommendations and published studies about the relevant indications and skills for the use of POCUS in primary care (general practitioners and emergency physicians) covering the abdominal and digestive, gynecological and obstetric, urogenital and vascular fields. We used various scientific databases PubMed® and Google Scholar®. The Medical subject headings (MeSH) terms used were: "general practitioner", "primary care", "family practice", “ultrasonography”. To precise our search, we used the following keywords: "clinical ultrasound", "POCUS", "Point-of-Care Ultrasound", "bedside ultrasound". A synthesis was produced and a list of skills, as exhaustive as possible, was drawn up by two co-authors LC and MS. The experts did not take part in this process. During the Delphi process, the experts were asked to rate each skill according to the 9-point Likert scale, from 1 “totally inappropriate skill” to 9 “totally appropriate”. Strong appropriate agreement was defined as 100% of “7–9” ratings. Strong inappropriate agreement was defined as 100% of “1–3” ratings. Relative agreement was defined as 100% of “1–5” or “5–9” ratings. We communicated with the experts by email and suggested they attend an information meeting. At each stage, the questionnaire was proposed via the LymeSurvey® online software.

#### Delphi round

The research team sent each expert the first questionnaire, in which they should rate and comment on each skill. They were allowed to propose new skills (not featured on the list provided).

#### Intermediate meetings

Between the two questionnaires, two successive intermediate meetings were organized one month apart. Each meeting was recorded, and a summary was produced. The skills that obtained a strong agreement (appropriate or inappropriate) in the first questionnaire were not discussed. The aim was to return the anonymized answers from the first round to the experts and discuss each non-consensual item and their respective answers.

#### Delphi round 2

A second questionnaire was created based on the initial list of skills with the addition of the changes suggested by the experts. It was sent to the experts with a summary of the discussions held at the intermediate meetings. The aim was to obtain a final consensus.

Data processing was done on a secured platform according to *French General Data Protection Regulation*. This project was conformed to *the National Commission of Computing and Freedom* and submitted to the Data Protection Officer of *Sorbonne University.* The protocol of the study was submitted to the Ethics committee of Sorbonne University. The Ethics committee answered that there is no need to seek ethical advice for work that relies on a Delphi round consensus method among professionals.

## Results

**Fig. 1:**
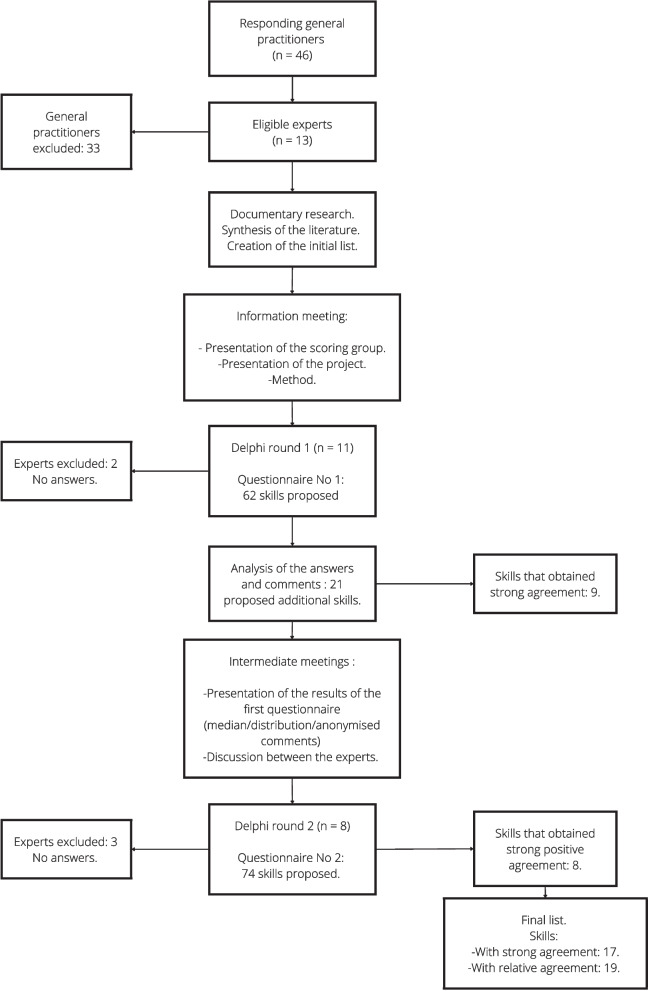
Study flowchart *(n* = *number of experts)*

Forty-six positive responses by general practitioners interested in participating in this study were received. Thirteen general practitioners were eligible and 11 responded to the first questionnaire (Fig. 1). A summary of the characteristics of the general practitioners experts in POCUS is given in Table 1**.** Nine of the 11 experts (82%) were ultrasound instructors. The two experts who were not instructors had more than five years of experience in POCUS.

**Table 1:** Characteristics of the experts. *(n* = *number of experts)*

	*n* =	%
Gender		
Male	7	64
Female	4	36
Age		
30–39	4	37
40–49	3	27
50–59	1	9
60–69	3	27
Department of practice		
Paris (75)	4	37
Haute-Garonne (31)	1	9
Pyrénées-Atlantiques (64)	1	9
Bas-Rhin (67)	1	9
Lozère (48)	1	9
Seine-et-Marne (77)	1	9
Yvelines (78)	1	9
Vaucluse (84)	1	9
Type of practice		
Group practice	4	37
Multidisciplinary healthcare facility (MSP)	3	27
Healthcare centre	1	9
Substitute in a group practice	1	9
Associate in MSP	1	9
Mixed: Hospital and MSP	1	9
Theoretical training		
Yes	11	100
*University training (degree/interuniversity diploma/*etc*.)*	*3*	*27*
*Continuous Professional Development*	*2*	*18*
*Mixed*	*6*	*55*
No	0	0
Length of ultrasound practice		
Between two and five years	2	18
Between five and ten years	5	46
Over 10 years	4	36
Ultrasound instructor		
Yes	9	82
*University training*	*1*	*9*
*Private training*	*5*	*46*
*Mixed*	*3*	*27*
No	2	18
Equipped with an ultrasound device		
Yes	11	100
No	0	0
Types of ultrasound practised		
Abdominal	11	100
Renal-urinary tract	11	100
Soft tissue	10	91
Testicular	10	91
Thyroid	10	91
Gynaecological	9	82
Vascular	9	82
Pulmonary	9	82
Muscular-skeletal	7	64
Cardiac	6	55
Obstetric	4	36
Ophthalmic	2	18

The first questionnaire included 62 skills divided into: 16 abdominal and digestive items, 19 gynecological and obstetric items, 13 urogenital items and 14 vascular items. The first Delphi round resulted in 9 skills with strong agreement (Table 2).

**Table 2:** Skills list (9) that obtained strong appropriate agreement after the first

Skills	Median	Distribution
**Abdominal/digestive**		
Affirm or not the presence of biliary lithiasis(es)	9	8–9
Affirm or not elements indicating cholecystitis	9	8–9
Affirm or not the existence of a peritoneal effusion	9	9–9
**Gynecology/Obstetric**		
Identify the precise location of an intrauterine device	9	9–9
Affirm or not a viable intrauterine pregnancy	9	8–9
**Urogenital**		
Affirm or not pyelocaliceal cavity dilatation	9	8–9
Measure urinary bladder volume	9	8–9
Affirm or not post-micturition residue	9	9–9
**Vascular**		
Affirm or not an abdominal aortic aneurysm over 5 cm	9	7–9

Delphi round,classified according to the skills fields studied. Strong agreement is obtained if the distribution is between “1–3” or “7–9”

The responses of the first round included 21 supplementary skills proposed by the experts. The supplementary skills were added to the second questionnaire, composed of 74 skills. The intermediate meetings were summarized. In total, 83 POCUS skills (Appendix 1) were screened.

The second round resulted in 8 skills with strong agreement and 19 skills with relative agreement**.** Three of the 11 experts did not answer the last questionnaire. Table 3 shows the final list of the 17 skills that obtained a consensus with strong agreement and the 19 skills that obtained relative agreement including 4 skills with inappropriate agreement. The number of experts who rated each skill for which a consensus was obtained is available on Table 3.

**Table 3:** Final skills list (36) that obtained a consensus after two Delphi rounds classified according to the skills field studied

Skills	Median	Distribution	Agreement		Exp.
**Abdominal/digestive**					
Affirm or not the presence of biliary lithiasis(es)	9	8–9	Ap	Strong	11
Affirm or not elements indicating cholecystitis	9	8–9	Ap	Strong	11
Affirm or not the existence of a peritoneal effusion	9	9–9	Ap	Strong	11
Differentiate between a healthy appendix and a pathological appendix	7	5–9	Ap	Relative	8
Identify mesenteric adenitis	7	5–9	Ap	Relative	8
Affirm or not dilatation of the common bile duct (hepatic duct and bile duct)	9	6–9	Ap	Relative	8
Suggest hepatic cirrhosis when hepatic dysmorphia is found	7	5–9	Ap	Relative	8
Affirm or not the presence of a focal liver lesion	8	5–9	Ap	Relative	8
Affirm or not constipation in children	2,5	1–5	Inap	Relative	8
**Gynecology/Obstetric**					
Identify the precise location of an intrauterine device	9	9–9	Ap	Strong	11
Affirm or not a viable intrauterine pregnancy	9	8–9	Ap	Strong	11
Assess gestational age of an intra-uterine pregnancy	9	5–9	Ap	Relative	8
Evoke an ectopic pregnancy	9	5–9	Ap	Relative	8
Affirm or no intrauterine fibroids	7	5–9	Ap	Relative	8
Affirm or not the presence of a subcutaneous contraceptive implant	9	5–9	Ap	Relative	8
Estimate fetal weight, in the second or third trimester of pregnancy	1	1–5	Inap	Relative	8
Affirm or not adnexal torsion	2,5	1–5	Inap	Relative	8
Affirm or not a sufficient quantity of amniotic fluid, in the second and third trimester of pregnancy	1	1–5	Inap	Relative	8
**Urogenital**					
Affirm or not pyelocalyceal cavity dilatation	9	8–9	Ap	Strong	11
Affirm or not elements suggesting urinary lithiasis	8,5	7–9	Ap	Strong	8
Affirm or not a bladder mass	9	8–9	Ap	Strong	8
Affirm or not the presence of bladder diverticula	9	7–9	Ap	Strong	8
Affirm or not a bladder globe	9	9–9	Ap	Strong	8
Measure urinary bladder volume	9	8–9	Ap	Strong	11
Affirm or not post-micturition residue	9	9–9	Ap	Strong	11
Measure post-micturition residue	9	8–9	Ap	Strong	8
Affirm or not a hydrocele	9	7–9	Ap	Strong	8
Affirm or not a testicular mass	9	9–9	Ap	Strong	8
Measure the bladder wall thickness	8,5	5–9	Ap	Relative	8
Affirm or not a retentional bladder	7	6–8	Ap	Relative	8
Measure prostate volume	8,5	6–9	Ap	Relative	8
Affirm or not a varicocele	7	6–9	Ap	Relative	8
Affirm or not elements suggesting epididymo-orchitis	7,5	6–9	Ap	Relative	8
**Vascular**					
Measure the diameter of the abdominal aorta	9	8–9	Ap	Strong	8
Affirm or not an abdominal aortic aneurysm over 5 cm	9	7–9	Ap	Strong	11
Affirm or not a proximal Iliac artery aneurysm	7,5	5–9	Ap	Relative	8

Inappropriate agreement (*Inap.*) is obtained if the median is “1–3”, appropriate agreement (*ap.*) is obtained if the median is “7–9”

Strong agreement is obtained if the distribution is “1–3” or “7–9”; agreement is relative if the median is “1–5” or “5–9”

Exp. number of experts participating in the 1st Delphi round (= 11) and 2nd Delphi round (= 8)

## Discussion

Using the Delphi method, this study made it possible to create a list of relevant skills in POCUS that are useful to general practitioners: this includes 36 skills that obtained expert consensus in the abdominal, urogenital, vascular, gynecological and obstetric fields (Table 3).

### Strengths and limitations

This study is to our knowledge the first French general practice consensus on POCUS skills in abdominal, urogenital, vascular, gynecological and obstetrical fields.

The number of experts included is in line with the literature [12, 13]. They had solid practical experience: 80% of them had been practicing for more than five years or were ultrasound instructors.

The proposals from the first questionnaire were the result of a synthesis of two types of publication:

- international publications that made it possible to obtain a list of indications in General Practice or which assessed the use of targeted ultrasound in some indications [3, 5, 14, 15].
- the recommendations of French-speaking and international learned societies such as the *AAFP* [4], the *French Society of Emergency Medicine* [16, 17], the *French Good Practice Guidelines for the Clinical Use of Medical Imaging* and the *French Guidelines for Requesting Radiology examinations and Medical Imaging* [18, 19].

With the skills proposed by the experts, 83 items were assessed (Appendix 1). We decided to conduct two Delphi rounds to reduce the attrition bias and a bandwagon effect [20]. Any authority bias was limited by making the answers anonymous and giving space to experts to comment on each skill. For a consensus, we required 100% of the highest scores (“7–9”); consequently, the consensus for each validated skills was total between the experts.

We faced difficulties in organizing the intermediate meetings: only three to six experts were present at the same time. Three of the 11 experts did not finish the process. This could affect the comprehensiveness of the final list so we precised the number of experts who rated each skill for which a consensus was reached. External factors such as COVID-19 pandemic, the winter season, the workload of GPs working in private practices in a context of lack of GPs in France might have affected the feasibility and execution of the study. We suggest these factors should be taken into consideration before the execution of future studies.

In order to reduce the effect on the Delphi method of the 3 non-respondents during the 2nd round, we considered that a strong or relative agreement should include 100% of the participants.

A single disagreement resulted in a non-consensual skill. For example: the proposition "affirm or not splenomegaly" did not obtain agreement even though the median of the responses was “8” because one expert’s rating was “3”.

In a qualitative study, « transferability» replaces the notion of external validity [21]. Our study cannot be generalized to all general practitioners. It would be interesting to interview « non-expert» physicians on the possibility of the use of this list of skills in common practice [10]. The non-expert perspectives might contextualize the need for certain ultrasound skills and describe different needs according to their type of practice and specific interest. Some skills that did not obtain consensus and were left out might be questionable in the context of a remote setting where the waiting time for an ultrasound is long. An observational study describing the use and impact of POCUS in real-world settings might provide further insights into the acceptance of the identified skills [11]. The consensual list could be compared to the variety of the actual skills used, the type and frequency of organs scanned.

### Discussion of the results

This study is a response to the prerequisite set by the *French Health Authority* [7] by identifying relevant skills in routine general practice, making it possible to specify the clinical situations in which POCUS is useful or POCUS training methods.

The *AAFP* work identified the basic applications, which include most of our final list, and the more advanced applications, such as searching for urinary lithiasis and hydroceles, which both obtained strong agreement in our study. The overlap was assessed for each field: 80% for obstetrical applications (first trimester), 25% for gynecology, 57% for abdominal (100% basic skills), 67% for urogenital and 67% for arterial applications in the vascular field. The skills proposed by the AAFP in 2017 overlap in total by 36% (19/53) with our list, an apparently low rate but a discordance is mainly due to the inclusion of obstetrical skills in the 2nd and 3rd trimester of pregnancy. In France, GPs play a role in the diagnosis of early pregnancy, in the follow-up of non-pathological pregnancies and in the voluntary interruption of pregnancy. They do not perform obstetrical deliveries. The experts focused on the value of POCUS before the 11th week of amenorrhea. The 2 skills that received an inappropriate agreement illustrate this ("estimate fetal weight, in the second or third trimester of pregnancy" and "affirm or not a sufficient quantity of amniotic fluid, in the second and third trimester of pregnancy"). Some skills are not mentioned, such as searching for bladder masses, bladder diverticula and testicular masses [4]. During the discussions, the experts pointed out that bladder diverticula were often a consequence of urinary tract obstruction and could be the reason for repetitive urinary infections. The “affirm of not the presence of bladder diverticula” was added to other skills that received strong agreement in the urogenital area like "affirm or not post-micturition residue" or "measure prostate volume". The experts pointed out that diagnosing complications of bladder diverticula such as urinary tract infections or stones was common in general practice.

Løkkegaard et al. obtained a list of 30 POCUS skills in general practice from Scandinavian general practitioners. In the 4 fields discussed in this study, the final list overlaps theirs by 85%. Some of our skills were not retained in their final list, such as searching for testicular and bladder masses. Others were not mentioned, such as searching for urinary lithiasis, bladder diverticula, and assessing post-micturition residue [5]. The 12 skills concerning with musculoskeletal ultrasound were not investigated in our study. The diagnosis of deep vein thrombosis was not included in our study because of a disagreement over the technical methods used (full ultrasound examination or point-by-point examination). Conangla-Ferrin et al. conducted a study similar to our study in 2022 by the *Catalan society of Family and Community Medicine.* Our results overlap 50% of the skills described in their final list. The skill mentioning appendicitis got only relatively agreed in our study. The gynecological applications were not very detailed [6]. The assessment of pancreatitis was not proposed in our initial list and was not added by the experts after the 1st round.

The differences in the composition of the expert group that rated the skills and the participants in the intermediate meetings might explain some of the discrepancies observed. Some skills were considered as advanced (e.g. appendicitis) or too specialized (assessment of pancreatitis) and did not obtain consensus. In the Spanish study, other specialists than general practitioners, as radiologists, participated in intermediate meetings. We do not know the level of expertise of the GPs in the Spanish study. But the participation of other specialists than general practitioners in the intermediate meetings might influence participants to include more advanced objectives and skills.

Another reason for discrepancies might be the different rating methods. In the Spanish study, the 75th percentile limit for obtaining consensus might have enabled a larger list to be obtained.

In France, the French Society of Emergency Medicine issued recommendations on clinical ultrasound skills in emergency medicine according to 2 skill levels that include part of our list [16, 17].

The skill « affirm or not the presence of bladder diverticula» proposed by one of the experts and which obtained strong agreement, is not mentioned in these various publications.

Sorensen et al. conducted a literature review of the clinical studies assessing POCUS in relevant indications in General Practice. Most of the publications focus on emergency doctors and not general practitioners [22]. Lindgaard et al. did assess general practitioners and found satisfactory results for the following skills: biliary lithiasis (se. 92% and sp. 92%), ascites (se. 100%, sp. 100%), abdominal aortic aneurysm (se. 100%, sp. 100%), developing intrauterine pregnancy (se. 100%, sp. 100%) [23]. Esquerrà et al. found notably similar results for the detection of biliary lithiasis by general practitioners [24]. Bravo et al. [25], Blois [26] and Bailey et al. [27] arrived at the same conclusions for abdominal aortic aneurysms. Nixon et al. assessed general practitioners in rural hospitals in New Zealand in renal POCUS and found good performance in relation to hydronephrosis (se. 90%, sp. 96%) and urine retention (se. 100% and sp. 100%) [28].

### Perspectives

Echoing the proposals made by the *French Health Authority*, the experts raised the need to conduct research enabling study, to a sufficient standard of proof, of the diagnostic performance of POCUS associated with a clinical examination, versus a clinical examination alone, in addition to assessing POCUS versus a reference test [29].

It seems crucial to provide details of the modalities for each skill: the corresponding clinical situations (e.g. suspect cholecystitis in the presence of acute, continuous abdominal epigastric pain increased by deep inspiration), the expected impact on decision-making in the patient’s care (e.g. referral to an emergency service, to specialist radiologists or only reassurance), the conditions required to achieve this act (e.g. stationary or ultra-portable device, type of ultrasound technology available such as B-mode, color Doppler, pulsed Doppler or the type of probe to be used or scanning technique) and its technical limitations (e.g. in cholecystitis, a gallbladder completely filled with stones can be difficult to see or stones located in the fundus or collum may therefore go unnoticed), as well as the learning outcomes for each skill (e.g. know the ultrasound characteristics of gallstones, know that the normal wall of the gallbladder is less than 3 mm thick) [30]. These details would make it possible to create support tools for the practice, such as the “action cards” proposed by the Danish Society for Ultrasound in General Practice, which are accessible from the Danish public health portal [31]. GPs using POCUS engage their own responsibility. Indeed, the experts raised the medico-legal risk that GPs are exposed to when they carry out POCUS (particularly in gynaecology/obstetrics). They stress the importance of explaining, in their clinical observations and to their patients, the impossibility of excluding a pathology in the absence of any visualised sign, or of specifying that "no morphological study has been carried out on this examination" (e.g. in targeted obstetric ultrasound during the first trimester of pregnancy). The description of the modalities helps physicians faced with the limitations of POCUS. By delimiting the field of reliability of POCUS exams, GPs will be encouraged not to perform POCUS outside the indications by being informed of the possible risks (e.g.: delaying treatment due to a falsely reassuring ultrasound).

## Conclusion

This study made it possible to obtain a consensus concerning 36 POCUS skills relevant in General Practice in the digestive (3 strong agreement/6 relative agreement), urogenital (10 strong agreement/5 relative agreement), gynecological-obstetrical (2 strong agreement/4 relative agreement) and vascular fields (2 strong agreement/1 relative agreement). This list of skills provides a basis for future clinical guidelines and training programs by identifying useful skills for general practitioners. It also acts as a guardrail by distinguishing between appropriate and inappropriate skills in general practice: it enables GPs to use POCUS to reach safe conclusions for their patient by making faster and more targeted referrals, and for themselves from a medico-legal perspective. This consensual list is not exhaustive, since new indications and possibilities for semiological examinations are emerging, meaning that the list should be continuously updated. Further studies should include other areas of interest such as thyroid, musculoskeletal, cardiac, soft tissue and pulmonary ultrasound.

### Supplementary Information

**Additional file 1: Appendix 1.** List of 83 skills proposed in both Delphi rounds with the respective level of expert agreement, classified according to the skills field studied.

## Data Availability

The datasets used and analysed during the current study are available from the corresponding author on reasonable request.

## Abbreviations

POCUS: Point-Of-Care Ultrasound
AAFP: American Academy of Family Physicians
GP: General Practitioner
Inap: Inappropriate
Ap: Appropriate
