# Supplementary material for: Consensus on relevant point-of-care ultrasound skills in General Practice: a two-round French Delphi study
Source: BMC Med Educ. 2024 Mar 26;24:341. doi: 10.1186/s12909-024-05072-3 (PMC10967120; doi:10.1186/s12909-024-05072-3)
Supplement: Supplementary file 1 — Additional file 1: Appendix 1. List of 83 skills proposed in both Delphi rounds with the respective level of expert agreement, classified according to the skills field studied. [file 12909_2024_5072_MOESM1_ESM.docx]

Appendix 1: list of 83 skills proposed in both Delphi rounds with the respective level of expert agreement, classified according to the skills field studied.

*Inappropriate agreement is obtained if the median is “1-3”, appropriate agreement is obtained if the median is “7-9”.*

*Strong agreement is obtained if the distribution is “1-3” or “7-9”; agreement is relative if the median is “1-5” or “5-9”.*

| Skills | Median | Distribution | Agreement |
| --- | --- | --- | --- |
| **Abdominal/digestive** | | | |
| Affirm or not the presence of biliary lithiasis(es). | 9 | 8-9 | STRONG |
| Affirm or not elements indicating cholecystitis. | 9 | 8-9 | STRONG |
| Affirm or not the existence of a peritoneal effusion. | 9 | 9-9 | STRONG |
| Differentiate between a healthy appendix and a pathological appendix. | 7 | 5-9 | RELATIVE |
| Identify mesenteric adenitis. | 7 | 5-9 | RELATIVE |
| Affirm or not dilatation of the common bile duct (hepatic duct and bile duct). | 9 | 6-9 | RELATIVE |
| Suggest hepatic cirrhosis when hepatic dysmorphia is found. | 7 | 5-9 | RELATIVE |
| Affirm or not the presence of a focal liver lesion. | 8 | 5-9 | RELATIVE |
| Affirm or not constipation in children. | 2.5 | 1-5 | RELATIVE |
| Affirm or not the presence of complicated appendicitis. | 7 | 1-9 | - |
| Affirm or not hepatomegaly. | 5 | 1-9 | - |
| Affirm or not splenomegaly. | 8 | 3-9 | - |
| Recognise the signs of chronic hepatitis. | 4.5 | 2-6 | - |
| Affirm or not the presence of a digestive abdominal mass. | 5 | 2-9 | - |
| When an abdominal mass is found, identify a digestive hernia. | 7 | 2-9 | - |
| Identify a strangulated digestive hernia. | 6 | 1-9 | - |
| Affirm or not an occlusion of the small intestine or colon. | 5.5 | 1-9 | - |
| When presented with acute abdominal pain, identify an acute intestinal intussusception. | 6 | 2-9 | - |
| Know how to empirically quantify peritoneal effusion: low, medium or high abundance. | 8 | 3-9 | - |
| Affirm or not elements suggesting an inflammatory pathology of the intestine. | 4.5 | 1-7 | - |
| Identify sigmoid diverticulitis | 7 | 1-9 | - |
| Identify complicated diverticulitis. | 6 | 3-9 | - |
| Identify appendagitis. | 3 | 1-6 | - |
| **Gynecology/Obstetric** | | | |
| Identify the precise location of an intrauterine device. | 9 | 9-9 | STRONG |
| Affirm or not a viable intrauterine pregnancy. | 9 | 8-9 | STRONG |
| Assess gestational age of intrauterine pregnancy. | 9 | 5-9 | RELATIVE |
| Evoke an ectopic pregnancy. | 9 | 5-9 | RELATIVE |
| Affirm or not intrauterine fibroids. | 7 | 5-9 | RELATIVE |
| Affirm or not the presence of a subcutaneous contraceptive implant. | 9 | 5-9 | RELATIVE |
| Estimate foetal weight, in the second and third trimester of pregnancy. | 1 | 1-5 | RELATIVE |
| Affirm or not adnexal torsion | 2.5 | 1-5 | RELATIVE |
| Affirm or not a sufficient quantity of amniotic fluid, in the second and third trimester of pregnancy. | 1 | 1-5 | RELATIVE |
| Affirm or not a molar pregnancy. | 5 | 1-9 | - |
| Precisely identify the position of the placenta, in the second and third trimester of pregnancy. | 1.5 | 1-8 | - |
| Affirm or not a detached placenta, in the second and third trimester of pregnancy. | 1 | 1-8 | - |
| Affirm or not uterine cervical shortening, in the second and third trimester of pregnancy. | 1.5 | 1-9 | - |
| Precisely identify the foetal presentation, in the second and third trimester of pregnancy. | 1.5 | 1-9 | - |
| Measure foetal growth (abdominal circumference, biparietal diameter, femur length), in the second and third trimester of pregnancy. | 1 | 1-7 | - |
| Identify the sex of the foetus, in the second and third trimester of pregnancy. | 1 | 1-8 | - |
| Affirm or not cardiac activity at any stage of the pregnancy. | 8.5 | 1-9 | - |
| Affirm or not normal endometrium images. | 7 | 3-9 | - |
| Measure the endometrium according to the cycle. | 7 | 2-9 | - |
| Affirm or not the presence of a functional ovarian cyst. | 6.5 | 1-9 | - |
| Affirm or not the presence of a haemorrhagic ovarian cyst. | 5.5 | 1-9 | - |
| Affirm or not the presence of polycystic ovaries. | 5.5 | 1-9 | - |
| If a mammary mass is found, suggest a simple mammary cyst. | 7 | 1-9 | - |
| Affirm or not the presence of an intramammary abscess. | 7 | 4-9 | - |
| **Urogenital** | | | |
| Affirm or not pyelocaliceal cavity dilatation. | 9 | 8-9 | STRONG |
| Measure urinary bladder volume. | 9 | 8-9 | STRONG |
| Affirm or not post-micturition residue. | 9 | 9-9 | STRONG |
| Affirm or not elements suggesting urinary lithiasis. | 8.5 | 7-9 | STRONG |
| Affirm or not a bladder mass. | 9 | 8-9 | STRONG |
| Affirm or not the presence of bladder diverticula. | 9 | 7-9 | STRONG |
| Affirm or not a bladder globe. | 9 | 9-9 | STRONG |
| Measure post-micturition residue. | 9 | 8-9 | STRONG |
| Affirm or not a hydrocele. | 9 | 7-9 | STRONG |
| Affirm or not a testicular mass. | 9 | 9-9 | STRONG |
| Measure the bladder wall thickness. | 8.5 | 5-9 | RELATIVE |
| Affirm or not a retentional bladder. | 7 | 6-8 | RELATIVE |
| Measure prostrate volume. | 8.5 | 6-9 | RELATIVE |
| Affirm or not a varicocele. | 7 | 6-9 | RELATIVE |
| Affirm or not elements suggesting epididymo-orchitis. | 7.5 | 6-9 | RELATIVE |
| Affirm or not the presence of a renal mass. | 7.5 | 2-9 | - |
| Affirm or not the presence of a simple kidney cyst. | 8.5 | 2-9 | - |
| Affirm or not elements suggesting pyelonephritis. | 3.5 | 1-9 | - |
| Affirm or not renal artery stenosis. | 2.5 | 1-6 | - |
| Affirm or not benign prostatic hypertrophy. | 7.5 | 1-9 | - |
| Measure the prostatic protrusion index. | 2 | 1-9 | - |
| Affirm or not testicular torsion. | 5.5 | 1-9 | - |
| **Vascular** | | | |
| Affirm or not an abdominal aortic aneurysm over 5 cm. | 9 | 7-9 | STRONG |
| Measure the diameter of the abdominal aorta. | 9 | 8-9 | STRONG |
| Affirm or not a proximal Iliac artery aneurysm. | 7.5 | 5-9 | RELATIVE |
| Affirm or not a thoracic aortic aneurysm. | 4 | 1-9 | - |
| Affirm or not a thoracic aortic dissection. | 1 | 1-8 | - |
| Affirm or not an abdominal aortic dissection. | 7.5 | 3-9 | - |
| Affirm or not an aneurysmatic intramural thrombus. | 6.5 | 1-9 | - |
| Affirm or not carotid stenosis. | 6.5 | 1-9 | - |
| Affirm or not proximal deep venous thrombosis by two-point compression: common femoral vein and popliteal vein. | 9 | 1-9 | - |
| Affirm or not proximal deep venous thrombosis by three-point compression: common femoral vein, superficial femoral vein and popliteal vein. | 9 | 1-9 | - |
| Affirm or not proximal deep venous thrombosis by examination from the inferior vena cava to the popliteal vein including the arch of the great and small saphenous veins. | 6.5 | 5-9 | - |
| Affirm or not arteriopathy of the lower limbs. | 5.5 | 1-9 | - |
| Know how to locate arteriopathy lesions of the lower limbs. | 2 | 1-7 | - |
| Guiding venipuncture. | 4 | 1-9 | - |
